# A longitudinal study of attitudes toward evolution among undergraduates who are members of the Church of Jesus Christ of Latter-day Saints

**DOI:** 10.1371/journal.pone.0205798

**Published:** 2018-11-07

**Authors:** William S. Bradshaw, Andrea J. Phillips, Seth M. Bybee, Richard A. Gill, Steven L. Peck, Jamie L. Jensen

**Affiliations:** 1 Department of Microbiology & Molecular Biology, Brigham Young University, LSB, Provo, UT, United States of America; 2 Department of Biology, Brigham Young University, LSB, Provo, UT, United States of America; Fordham University, UNITED STATES

## Abstract

Polling data reveal a decades-long residual rejection of evolution in the United States, based on perceived religious conflict. Similarly, a strong creationist movement has been documented internationally, including in the Muslim world. Members of the Church of Jesus Christ of Latter-day Saints (LDS, Mormon), a generally conservative denomination, have historically harbored strong anti-evolution sentiments. We report here a significant shift toward acceptance, compared to attitudes 30 years earlier, by students at Brigham Young University, which is owned and operated by the LDS church. This change appears to have multiple explanations. Students currently entering the university have been exposed to a much-improved introduction to evolution during high school. More importantly, there has been a significant decrease in negative messaging from Church authorities and in its religious education system. There is also evidence that current students have been positively influenced toward evolution by their parents, a large percentage of whom were BYU students, who earlier were given a strong science education deemed compatible with the maintenance of religious belief. A pre-post comparison demonstrates that a majority of current students become knowledgeable and accepting following a course experience focused on evolutionary principles delivered in a faith-friendly atmosphere. Elements of that classroom pedagogy, intended to promote reconciliation, are presented. Our experience may serve as a case-study for prompting changes in acceptance of evolution in other conservative religious groups.

## Introduction

The research reported here comes against a background of two relevant national trends in the United States. The first emerges from data showing a decline in religion. The Pew Research Center reports that the percent of US adults who believe in god, pray daily, and regularly attend church services has modestly declined in recent years [1,2]. A prediction consistent with this circumstance is that approval of a deity-less-conception of the role of evolution in life’s history should increase. The second trend suggests an impulse in the opposite direction, the so-called “War on Science” [3,4]. Examples of this phenomenon–a rejection of compelling evidence generated by empirical science—include the popularity of efforts to introduce “Intelligent Design” (ID, a theology-inspired anti-evolution notion) into the biology classroom of the public schools [5,6], parents who refuse to vaccinate their children as a hedge against autism [7,8], and the refusal of significant numbers of Americans to accept the reality of climate change [9] in spite of overwhelming evidence to the contrary [10]. Persons espousing these or other similar views would be expected to also reject the evidence that supports an evolutionary explanation for living organisms, this being another example of the alleged unreliability of scientific inquiry in the face of deeply held religious, personal, or political beliefs.

The present study examines a particular subgroup of religious individuals who identify as Mormons, or members of the Church of Jesus Christ of Latter-day Saints (LDS). This population represents a useful test-case, given that is highly homogenous, highly conservative, and has a long-standing cultural bias against evolution, for how increased approval of this fundamental scientific principle might be achieved. Our data suggest that there has been a positive shift in attitudes toward evolution among LDS college students over the past two decades. To add context to our study, we consider, the recent history of acceptance of evolution, first in the US and then internationally, and the resulting challenges faced by teachers in the science classroom as they attempt to inform students who come to them with pre-conceived religious objections.

### Evolution in the US

The results of national surveys in the United States show a consistent suspicion or rejection of evolution as the explanation for the origin of human beings. A Harris Poll in 2013 [11] reported a 35% national belief in “Creationism,” which was essentially unchanged since 2005. In that same interval, belief in “Darwin’s theory of evolution” increased from 42% to 47%. A Gallup poll in 2014 [12] showed that over the past three decades (since 1982) a constant creationist view of human origins has prevailed. In a narrow range fluctuating between 40–47%, Americans have opted for the view that “God created human beings pretty much in their present form at one time within the last 10,000 years or so.” Of those individuals, 69% attend religious services weekly, and 57% have no more than a high school education. The pollsters state that “Many religious Americans accept creationism mostly on the basis of their religious convictions. Whether their beliefs would change if they became more familiar with evolution is an open question.” The results of a Pew Research Center poll conducted in 2013 [13] show the same general trends, although the creationist view is lower (33%). An explanation for the differences between the Gallop and Pew numbers based on differences in the language of the survey questions has been offered [14]. Pew also reported that among US states, Utah, (headquarters of the LDS Church) at about 30% acceptance, is third from the bottom (only Tennessee and Arkansas are lower; [15]). In contrast, a survey of scientists in the American Association for the Advancement of Science showed that 98% accepted the view that humans and other living things have evolved over time [16]. In a Pew Research center study of 2014, the data were divided by religious affiliation. Fifty-two percent of Mormons expressed rejection (second only to Jehovah’s Witnesses at 74%) in comparison to Mainline Protestants—30%, Catholics—29%, and Jews—16% [17].

### The international perspective; Christian and non-Christian attitudes

An international survey of 34 countries in 2006 [18] determined responses to the proposition “Human beings, as we know them, developed from earlier species of animals”; the US ranked 33^rd^ in acceptance (only Turkey was lower). By comparison, about 80% of people in Denmark, Sweden, France, and Japan accepted the statement as true. A review published a year [19] later outlined the threat of creationism to biology education in Europe and South America. Academic science standards were being challenged in the UK by those espousing Biblical literalism. Similar campaigns were reported in Germany, France, Poland, Belgium, The Netherlands, and Scandinavia. In Latin America, only Brazil reports an anti-evolution movement like that in Western Europe. The message of a widely circulated, highly illustrated creationist text produced in Turkey [20] is summarized in the following quotation: “Fossils reveal that life forms on earth have never undergone even the slightest change and have never developed into one another.” This is part of a body of evidence suggesting that the Muslim world will be the next significant battleground in the conflict between religion and evolution [21].

### Dealing with religion in the science classroom

In an effort to understand how classroom experiences may affect our LDS population, we briefly review the important efforts, independent of the issue of religion, to identify effective pedagogical strategies for teaching science. One influential earlier report, for example, cited the importance of dealing with students’ preconceptions about how the world works, and promoting a metacognitive approach to instruction [22]. Alters and Nelson [23] focused on the first of these themes in the specific context of teaching evolution, by identifying misconceptions such as that mutations are always detrimental or that all individuals in a group evolve over time. These authors also advocated the used of active learning techniques such as pair-share discussion and the insertion of concept maps. Reiss [24] questions the utility of an either/or approach in teaching students of faith, and recommends a very modest expectation: “When faced with individuals who hold creationist views, science educators might be best advised to see creationism not as a naïve misconception but as a worldview, in other words, a fairly robust (durable) and well established (well defended) mental structuring of reality. The most that a science teacher can normally aspire to is to ensure that students with creationist beliefs understand the scientific position. In the short term, the scientific worldview is unlikely to supplant a creationist one.” Advocating a more proactive strategy, Calver and Bryant [25] have recently provided a useful proposal featuring a listing of diverse creationist positions, a review of responsive educational approaches, readings and resources for both teachers and students, and examples of effective classroom learning activities. Barnes and Brownell [26] have also offered an approach that utilizes cultural competence strategies to bridge the gap between a largely non-religious science professorship and a largely religious student audience.

### The LDS context

In specifically dealing with our study population, we present the LDS context. There is an extensive literature addressing the history of LDS views about evolution [27–29]. An important conclusion to be drawn from all the existing information is that the LDS Church has no official statement on the theory of evolution. In spite of this, several influential authorities have expressed anti-evolutionary opinions [30–32] that have imprinted several generations of Mormons with highly negative views about the subject, creating a cultural memory with strong staying power.

The Church’s flagship university, Brigham Young University (BYU), has been the venue for divergent ecclesiastic and academic views competing with each other on the subject of evolution. In the last half of the 20^th^ century, well publicized condemnatory messages were delivered to that student body by certain Church General Authorities [33, 34], reinforcing similar positions espoused by some teachers of religion courses [35, 36]. On the other hand, the validity of the relevant scientific research data and its compatibility with religious faith have been expressed during course instruction and in published papers by faculty in botany [37], geology [38], genetics [39], molecular biology [40], and ecology [41]. Something of a moratorium on overt conflict on this campus was achieved in 1992 with the production and acceptance of the “BYU Evolution Packet” ([42]; available at http://biology.byu.edu/ > Undergraduate Resources > Evolution Packet), a modest collection of relevant statements made by the Church’s First Presidency (“the definitive source of official Church positions”). (See additional details about the context and content of the “Packet” provided by Bailey [30]). The cover letter to this collection emphasizes the LDS doctrinal position on man as the spiritual offspring of deity, but clearly states, “There has never been a formal declaration from the First presidency addressing the general matter of *organic evolution* as a process for development of biological species.” We have gathered data via national polling showing evolution acceptance in various religious groups [43], and confirmed the LDS results in a preliminary study of BYU undergraduates [44].

For members of the LDS Church, then, there has been a discrepancy between an official statement, of limited circulation, that there is no doctrinal objection to biological evolution, and a widely-disseminated set of opinions by some high-placed church authorities condemning evolution. The present study, therefore, presents an important example of how greater acceptance of this foundational scientific principle might be achieved in the face of competing viewpoints in a highly conservative community.

### Research objectives

We had four research goals in the present study. First, we report our efforts to confirm a longitudinal shift toward acceptance of the tenets of biological evolution by current LDS students. Second, we test several possible explanations for it: A) Instruction in the introductory junior high and high school biology courses in which college-bound students have enrolled has improved, especially in exposure to and treatment of evolution; students entering the university are better acquainted with the facts and arguments, and hence better able to accept the validity of the subject; B) Anti-evolution pronouncements by Church authorities have decreased in number and intensity in the past 30 years; younger persons in the LDS community have less reason to reject the science based on religious objections; C) A significant proportion of students matriculating at BYU are the children of BYU graduates; acceptance of evolution due to information and attitudes acquired by parents during their BYU years in the preceding generation have been transmitted to their children; efforts by biology instructors at BYU help facilitate the reconciliation of science and religion such that evolution no longer poses a threat to an academically trained population within Mormonism. Third, we attempted to directly validate the positive impact of strategic instruction on student attitudes toward evolution based on a reconciliation model. This was accomplished using pre-post tests across a semester of introductory biology in two cohorts over a thirty-year longitudinal time period. Fourth, we explore the question of whether or not this shift in acceptance of evolution is a valid indicator of beliefs about the trustworthiness and utility of science in general.

## Materials and methods

### Ethics statement

Brigham Young Institutional Review Board for Human Subjects approved this research and granted permission for human subjects use in this study; written consent was obtained from all participants. All anonymous data are freely available at https://scholarsarchive.byu.edu/data/6.

### Cohort 1, 1986–1996

Data for Cohort 1 was collected from an introductory non-majors biology course. Biology 100 is a 3-credit hour required course in the general education program at BYU. Majors in biology-related departments taking a separate introductory course were exempted. Fifty-three percent were women. Fifty-seven percent were in the age range from 15–18 (freshmen), and 40% from 19–25 (upper-class persons). In Cohort 1 there were often 2 large sections accommodating 600–700 students each. The total enrollment across all sections was about 2,000 per semester (at that time there were fewer alternative courses that met the university general education requirement). From 1982–1996 (17 semesters in 12 years) a particular course content and scientific philosophy (see below) was taught by the same two instructors, one of whom is a present author (WSB), to 15,000 students. All the data reported here for Cohort 1 were collected from more than 14,000 total students during a somewhat shorter period when there was a uniformity of course assessments (1986–1996, 8 years, 12 semesters).

### Cohort 2, 2014–2016

Data for Cohort 2 was collected from the same introductory course, Biology 100. Total enrollment in these semesters was 1600–1700, in approximately 12 sections of 30–200, each taught by different instructors, one of whom is a present author (JLJ). The course enrollment typically consisted of 58% freshman, 25% sophomores, 11% juniors, and 6% seniors. Fifty-two percent were women. Fifty-six percent were in the age range from 15–18, and 41% from 19–25, with an average of 20 +/- 5.25 years.

### Course design

There are 42 class periods in a semester. In the earlier period (Cohort 1) two introductory periods focused on scientific methodology and data analysis, and ten periods were devoted to each of four major units, the third of which was Evolution The last two class periods of each unit were devoted to an exploration of an Application Issue, an ethical or religious concern–often controversial–related to the topic of that Unit (genetic engineering, reproductive technologies, evolution, and protection of the environment). In the evolution unit, the Application Issue was devoted to an attempt to help students reconcile their religious beliefs with the science. Among the course assignments administered at that time were sets of survey items described below (Data Collection) as Instruments 4 and 5. They were designed by one of us (WSB) with the intent to provide students with feedback about their own attitudes toward the science they were studying and its applications.

Data obtained from 2014–2016 (Cohort 2) came from multiple sections taught by different instructors, each using a somewhat unique course design. However, each section was taught a full unit on evolution consisting of the typical sequence of topics (e.g., evidences including fossils, vestigial traits, common genetic code, etc.; the processes of evolution, including natural selection, genetic drift, gene flow, non-random mating, and mutation; human evolution). In addition, each section began that unit with a one-class-period discussion aimed at reconciling evolution with religious beliefs including a discussion of the official church stance on human origins via the “BYU Evolution Packet” and a time for students to ask questions and discuss.

### Data collection

To address our first research questions (the documentation of a shift in acceptance over a 30-year time period), we administered a 500-word personal essay outlining one’s view of the meaning of evolution, before the presentation of that subject in class (See the instructions presented to students for this assignment, as S1 File). In addition, an 8-item questionnaire focused on specific issues related to evolution was administered at the end of the semester. To address our second research question, to explore potential reasons for the shift in a somewhat preliminary manner, we administered a pre-instructional survey whose purpose was to determine some of the relevant experiences of incoming students before instruction began. This survey was only administered to Cohort 2. The items included information on high school education, family circumstances, and LDS educational experience. To address our third research questions, to test the effectiveness of classroom instruction focused on reconciliation on student acceptance of evolution, a second 500-word essay was administered, written after the in-class presentation of evolution, that revisited the first essay and offered an explanation for any changes in attitude that might have occurred (see S2 File). To address our final research question, to investigate whether a shift in acceptance of evolution is an indicator of beliefs about the trustworthiness and utility of science in general, we included four questions on the already mentioned pre-instructional survey addressing issues related to other controversial scientific issues. In addition, we administered an 8-item questionnaire focused on other scientific issues and on the conflict between science and religion, post instruction, to both Cohort 1 and Cohort 2. Lastly, a 12-item questionnaire focused on specific issues related to ecology and the environment was administered at the end of the semester. These items serve as an internal control that helps validate the evolution data.

The survey of recent students (2014–2016) was approved by the institution’s IRB (study number E16323). All data is available at https://scholarsarchive.byu.edu/data/6.

### Creation and validation of survey instruments

The 8-item evolution questionnaire, 8-item scientific issues questionnaire, and 12-item ecology and environmental questionnaire were all created by original researchers during Cohort 1. They were validated by expert review, but no other validation was undertaken. In an effort to preserve a direct comparison of cohorts, we did not alter any of these survey items.

The pre-instructional survey and additional four items on controversial scientific topics that were only administered to Cohort 2 were created by researchers based on a deep understanding of their audience and the common issues with the culture (as all researchers are a member of the same culture, being members of the affiliated church). Experts validated the items based on their own experiences with students and student feedback. No additional validation was undertaken as the surveys were originally meant to be an exploration of potential causal factors. As such, we are presenting the results of these surveys as potential factors to be further investigated.

### Coding and inter-rater reliability of essay narratives

Essays were analyzed using thematic analysis with a constant comparison methodology [45] by twenty independent coders for Cohort 1 and thirteen independent coders for Cohort 2. Pre-essay and post-essay coding themes were established 30 years ago using an analysis of essays from Cohort 1. Cohort 2 data was fit to the themes, although new themes could emerge. Essays almost always included multiple themes, but were coded by selecting the single dominant theme that comprised the majority of the narrative, in an effort to not over-inflate the presence of the different attitudes. The pre- and post-coding themes are shown in S1 and S2 Files.

Cohort 2 coders were trained by one of the independent coders from Cohort 1. The training session included sample essays on which coders could practice. Cohort 2 coders were then given 30 essays to code independently after which coders got together to discuss and resolve differences. (A similar practice protocol was followed during the Cohort 1 period.) Agreement between the consensus code chosen and the original codes assigned was calculated as a percent agreement. This was averaged over the 30 essays. Coder agreement was low (68%). Coders were given 10 more essays to code. Interrater agreement of these additional 10 was 75%. A second discussion took place, after which coders were given an additional 25 essays to code; interrater agreement for these was 81% and deemed satisfactory, given that multiple themes may be present in a single essay. One additional post-essay code emerged from the data from Cohort 2 (I got substantial evidence to support my acceptance) that was not present in Cohort 1.

### Statistical analysis

Quantitative survey data was analyzed using the R statistical package [46]. Raw frequencies were calculated from the compiled data. Chi-squared goodness of fit analyses were run to compare differences in frequency distributions. If sample sizes in any category were sufficiently low to add potential error to the Chi-square calculation, a continuity correction was applied using 10,000 replicates. In this case, corrected values are reported. Coded essays were compared in similar fashion.

## Results

### Research Question 1: Has a longitudinal shift toward acceptance of evolution occurred over a 30-year time period?

#### Pre-instruction essay

**Fig 1:**
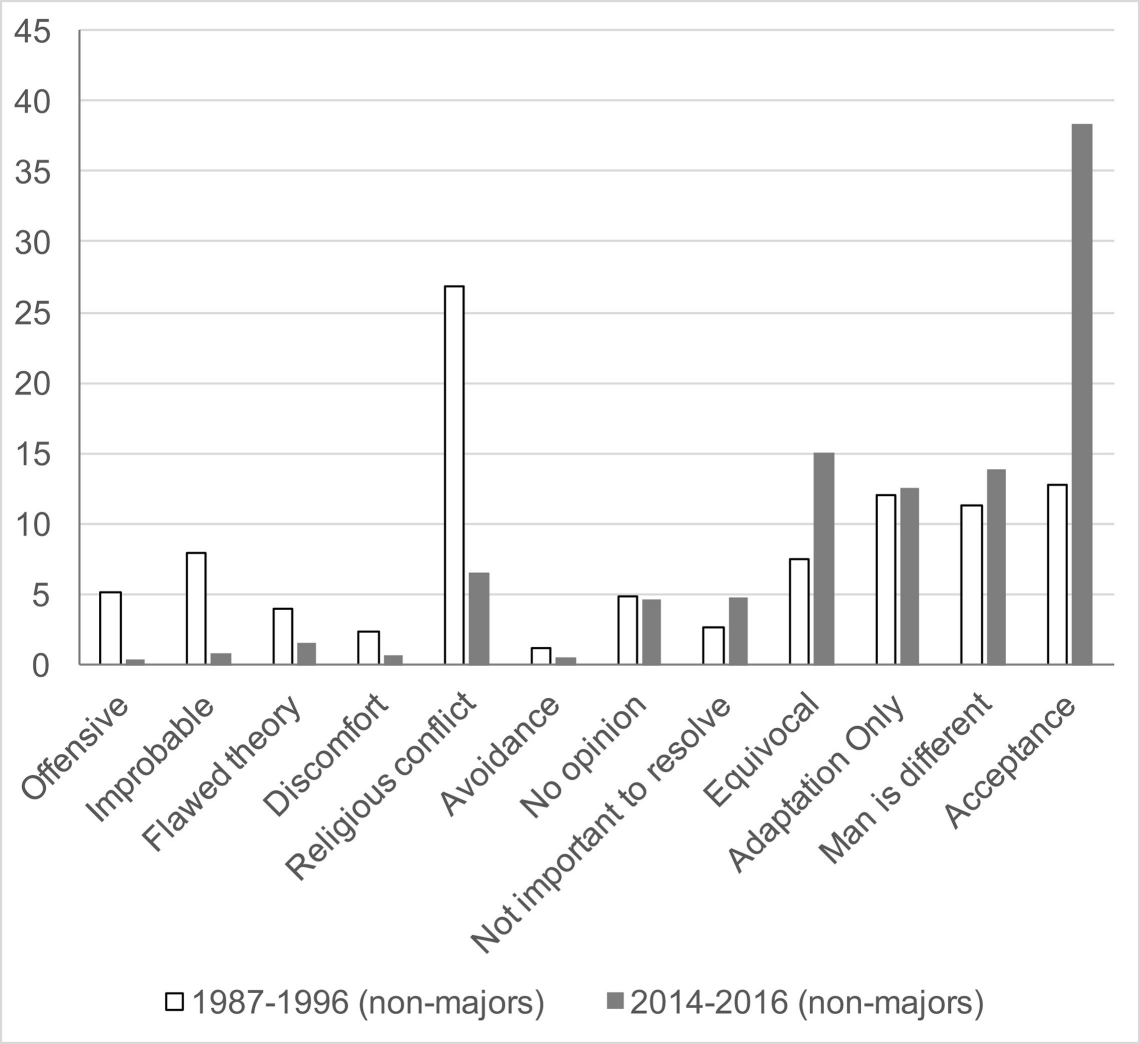
Pre-essay comparison in non-majors biology. This is a comparison of the distribution of essay themes in the non-majors biology courses (Biol. 100) between 1987–1996 (light bars) and between 2014–2016 (dark bars). Themes are abbreviated and ordered, approximately, from least accepting (left) to most accepting (right).

In order to answer our first research question and confirm a longitudinal shift in evolution acceptance, students in both Cohorts submitted a 500-word personal opinion piece (Pre-Instruction Essay) explaining their current understanding of the concept (see S1 File) early in the semester, before the evolution section of the curriculum was introduced. Each essay was coded by identifying the most prominent theme it contained (see the list in the coding rubric, S1 File; many essays contained multiple elements). The distribution and comparison of views between Cohorts is shown in Fig 1. The distributions are statistically significantly different, X^2^(11) = 40.90, *p* < 0.001. In the earlier period (Cohort 1), there was a significant diversity of initial reactions. Overall, however, there was a strong negative sentiment. Consider the following example;

“The first thoughts that this theory brings to my mind provoke a feeling of disgust and disagreement. This belief upsets me a great deal because of the passage in *Genesis* that states that God made man in His own image, and thus we are not some creature empirically derived from simpler life forms thought time. Evolution is nothing more to me than a thoughtless idea crated [sic] to explain how man came to be. Evolution makes me frustrated and upset; it has no direct application to today’s world.”

The largest single category (27%) expressed conflict with perceived LDS doctrine, with several other non-accepting rationales. Only 13% of the students expressed being comfortable with the scientific view.

In sharp contrast, the initial themes of the essays written by Cohort 2 students exhibit a 4-fold reduction in religious conflict with a 3-times greater level of acceptance or reconciliation with religious beliefs. For example,

“To me the concept of evolution makes sense. First off, there is ample evidence for it. How can we disregard something that has evidence? Evidence means truth, and God is truth. Now, just because science has proven that evolution happens, why should that mean that God was not involved in that process? The theory of Evolution is correct. God is behind it.”

There is another 15% (a 2-fold increase over Cohort 1) who find partial acceptance (“Equivocal” category), and approximately the same number who find compatibility with evolution with the exception of humans. For example,

“I believe that evolution, along with natural selection, is a real theory, and is true. However, I do not believe in the evolution of humans, including the idea that humans are just “more evolved apes”. I know that our species was created in God’s image, we are His children, and we have not changed for the thousands of years that we have been on this earth.”

About 12% of essays in both cohorts express acceptance of evolution as defined by adaptation to the environment, not as a process resulting in speciation.

In addition, we have observed that the contents of the recent essays clearly reflect improved coverage of evolutionary biology during the students’ K-12 preparation. Many offered correct descriptions of concepts like natural selection or made reference to canonical examples like Darwin’s finches. Conspicuously absent are significant expressions of repulsion, discomfort, or improbability in Cohort 2.

#### Post evolution survey

Important details about student views concerning evolution, both positive and negative, were obtained from the results of a questionnaire administered after the in-class coverage of the subject. These are shown in Table 1. Many of the major observations cited above (from written commentaries) are reinforced in these data. The 1986–1996 cohort had internalized some strong negative impressions about evolution; these were amplified as a result of the anti-evolution sentiments expressed concurrently by a high-ranking Church official (Packer, 34). In contrast, a very large shift toward acceptance is evident in the responses of the current cohort. The results for Question 1 show an almost negligible rejection due to religious conflict with a concomitant 39% increase in support of evolution for the present students (X^2^(5) = 31.02, *p* < 0.001). Ninety-two percent of this same group correctly supported the scientific definition for evolution as a theory (Question 2; X^2^(4) = 14.38, *p* = 0.06), whereas a large fraction of the earlier group viewed theory as an unproven assumption, although the shift failed to reach statistical significance. The Question 3 responses demonstrate the strong influence of authoritative institutional statements (27%) in 1988, following a campus-wide talk by a Church General Authority with a strongly-worded anti-evolution subject [34]. Clearly those remarks reinforced the perception of some persons of an official LDS position opposing evolution. This was a transient effect, however. In the semester following this event (Winter 1989), the data for this measure returned to the same level as for the semester before it took place (Winter 1987; data not shown). These data represent a reciprocal shift between Cohorts 1 and 2, with the influence of a secular approach increasing at the expense of a religious framework (Cohort 2 is equal to Cohort 1, overall; X^2^(4) = 6.60, *p* = 0.15). The most recent generation of students perceive themselves more informed about the subject (relative to an earlier generation) according to the results of Question 4 (X^2^(4) = 15.69, *p* = 0.005). Acceptance for a deep time estimate for the age of the earth increased by about 30% over the intervening 25 years (Question 5; X^2^(4) = 17.12, *p* < 0.001), with a 20% increase in acceptance of the validity of geological evidence (Question 6; X^2^(4) = 11.72, *p* = 0.01). A significant change (76% cf. 62%) occurred in students’ knowledge of the official position of the LDS Church (Question 8; X^2^(3) = 15.52, *p* = 0.001). About 20% of students in the earlier cohort responded with “None of the above,” an apparent indication of the unsettling uncertainty many of that time period felt about these issues. Finally, the results of Question 7 about the teaching of creationism in the public schools show a decreased approval of the “equal time” sentiment (X^2^(4) = 16.09, *p* = 0.001), but there is a lingering uncertainty about this issue, probably due to the partisan conservatism in the LDS community.

**Table 1:** Evolution questionnaire (percent responses) from each cohort.

Question	‘87 –‘96	Fall ‘88	2014–16
1. Which of the following best represents your overall personal view about the concept of biological evolution?			
a. It is a repulsive notion. I find it demeaning and degrading and resent the suggestion by misguided individuals that I am related to apes or single-celled organisms	2.0	4.0	0.5
b. I reject it as being illogical. It is too far-fetched to be believed. It is only a theory based on conjecture. When all the real facts are in, evolution will be disproved.	2.0	3.0	0.7
c. I reject evolution because it is in direct conflict with my religious faith. One cannot reconcile such a view of the origin and development of life with a belief in a divine creator.	8.6	16.0	2.0
d. Evolution might apply to some limited circumstances, but cannot be a general principle. (Evolution does not occur across the boundaries which separate the major categories of plants and animals. It may apply to lower forms, but not to man.	36.5	50.0	18.8
e. I accept the bulk of evolutionary ideas as true, but I don’t know how to reconcile them with religious concepts I also believe to be true. This is unsettling and I find myself confused.	27.5	16.0	17.5
f. I accept evolution as a true principle. There is strong evidence to support the concept, and I do not find it in conflict with my religious faith.	23.4	10.0	60.6
2. Which of the following represents your personal view about the scientific status held by evolution? What place does it hold in the hierarchy between absolute truth and falsehood?			
a. Evolution is a law, an absolute principle whose mechanisms and consequences are completely defined.	2.0	2.0	5.5
b. Evolution is a theory, meaning a concept consistent with a large body of evidence which best explains the diversity of life on earth and continues to stand the test of time and new data.	66.9	51.0	84.5
c. Evolution is a theory, meaning an unproven assumption or mental speculation. There is not yet enough evidence to consider evolution a valid principle.	28.0	43.0	9.0
d. Evolution is a postulate, a loose proposal about which there is little agreement among experts.	2.2	3.0	0.7
e. Evolution is a fraud. It is a deliberate misrepresentation of the truth without any basis in fact.	0.9	2.0	0.2
3. What or who has been the strongest influence on your in the formulation of your opinion about evolution?			
a. My parents	8.2	8.0	10.3
b. Sunday School/Seminary teachers, church leaders	13.7	27.0	4.8
c. Peers/friends	2.9	2.0	1.4
d. Courses/teachers in school	28.6	20.0	38.7
e. My own personal study and thought	46.6	43.0	44.8
4. How well informed are you on evolution? (To what degree have you studied the biological propositions and their philosophical consequences?) Place yourself on a scale from 1 (very well informed) to 5 (almost totally ignorant).	2.83	2.80	2.86
5. Which of the following best represents your personal view about the age of the earth?			
a. Relatively short (based on a literal interpretation of *Genesis*: each day of creation = 24 hours.	1.4	2.0	0.8
b. A few thousand years (based on an interpretation of scripture such that “1 day with God = 1,000 years with man”).	25.1	27.0	9.9
c. A few thousand years (based on the view that scientific dating techniques are inaccurate).	4.2	5.0	2.9
d. Very long, 4.6 billions years (based on the most widely accepted scientific estimate).	46.2	39.0	74.1
e. None of the above.	23.7	27.0	12.2
6. Which of the following best represents your view about fossils and other geological evidence which are cited in support of evolution?			
a. Such evidence is weak and not compelling. It is too fragmentary and incomplete to justify the speculative conclusions usually drawn from it.	16.2	25.0	4.8
b. Such objects were placed on the earth by God as a test of the faith of human beings.	4.3	6.0	4.0
c. Such objects were arranged on the earth by Satan in order to destroy the faith of human beings.	1.6	2.0	0.8
d. Such evidence is widespread and accurate and validates the concepts of evolution.	47.5	37.0	68.6
e. I have no opinion on the matter.	30.4	36.0	21.8
7. In June 1987 the United States Supreme Court ruled unconstitutional a Louisiana state law (The Balanced Treatment Act) which required that if evolutionary theory was taught in the public classroom, equal time must also be given to the presentation of creationism. Which of the following best describes your personal reaction to this decision?			
a. I approve. The teaching of fundamentalist religious ideas in the school would violate the principle of separation of church and state.	23.8	24.0	28.5
b. I disapprove. Evolution is an atheistic concept. To teach it in the public schools contributes to the breakdown of values I hold dear.	2.7	5.0	1.9
c. I disapprove. Equal time ought to be granted to opposing points of view on controversial issues.	49.1	48.0	23.7
d. I’m neutral. I’ve listened to both sides of the argument and don’t have strong feelings either way about the issue.	17.1	16.0	32.9
e. I haven’t paid any attention to this issue: it doesn’t hold much interest for me.	7.2	7.0	13.0
8. In your view, which statement below best represents the official position of the Church of Jesus Christ of Latter-day Saints toward the principle of biological evolution?			
a. The official position of the church is that evolution is incorrect. The idea is not in harmony with statements of the scriptures and church leaders, and is harmful to the spiritual growth of church members.	13.2	41.0	2.4
b. The official position of the Church is that evolution is correct. It is scientifically sound and compatible with the principles of the gospel.	5.2	3.0	12.1
c. There is no official position of the Church concerning evolution. A wide difference of opinion exists among both church leaders and members on the subject.	62.2	35.0	76.5
d. None of the above.	19.4	21.0	8.9

An additional column is included to show the effects of an influential talk by a Church authority given in Fall of 1988 (35). Following each question are the results of the Chi-square test of homogeneity comparing the distribution from Cohort 1 (1987–1996, N = 10,203) to the distribution from Cohort 2 (2014–2016, N = 646).

Overall, these data clearly demonstrate that the most recent students (Cohort 2) have a more positive view of evolutionary concepts than those a generation earlier. But in addition, the salutary influence of a strong classroom treatment of evolution is evident in both time periods.

### Research Question 2: Can we suggest potential explanations for this shift?

#### Pre-instruction survey–high school preparation and anti-evolution pronouncements

Information obtained from a survey administered early in the 2016 fall semester permitted us to test the first two suggested causal factors for a shift in acceptance. The data allowed for the formulation of a profile of student attitudes and experiences about evolution before the presentation of that subject in their university course (Table 2). (This survey was not administered in Cohort 1.)

**Table 2:** Views about, and experience with, evolution in a personal setting.

Question	Response (%)
1. What courses have you taken in high school? (Check all that apply.)	
a. Earth Science	36.2
b. General Biology	90.3
c. AP Biology	7.9
d. Specialty Biology (e.g. marine, environmental, etc.)	5.9
2. Did your high school biology course offer strong coverage of the topic of evolution?	
a. Strong coverage	31.4
b. Weak coverage	62.4
c. No coverage	6.2
3. Apart from covering the subject of evolution did your teacher reveal (perhaps inadvertently) a personal bias about evolution?	
a. A skepticism about the validity of evolution	4.2
b. A feeling that there might be a religious conflict about evolution	19.8
c. An atheistic attitude about the absence of deity in the evolutionary process	12.9
d. A neutral attitude; no bias about the consequences of evolutionary principles	63.2
4. What was your reaction to the topic of evolution as taught in your high school biology course?	
a. Acceptance without conflict	17.3
b. Concern about the validity of evolution	11.0
c. Feelings of mild conflict because of religious doctrine	30.0
d. Feelings of severe conflict leading to rejection of evolution	5.5
e. Feelings of neutrality; no strong reaction either way	36.2
5. Was evolution ever discussed in your LDS seminary classes?	
a. No	60.7
b. Yes. I came away with the feeling that the Church opposes evolution.	5.4
c. Yes. I came away with the feeling that the Church doesn’t have a position on evolution	4.0
d.Yes. I came away with the feeling that science, including evolution, and religion are compatible with one another.	23.5
e. Something else.	6.5
6. Have you heard about, or read, the book *Man His Origin and Destiny*, by Joseph Fielding Smith?	
Yes.	13.1
No.	86.9
7. Have you heard about, or read, the book *Mormon Doctrine*, by Bruce R. McConkie?	
Yes.	49.2
No.	50.8
8. Have you heard about, or read, the “BYU Packet on Evolution?”	
Yes.	9.5
No.	90.5
9. Have you recently heard or read a statement by a current LDS leader/authority which expressed negative sentiments about evolution?	
Yes.	8.1
No.	91.9
10. Was the teaching of evolution a controversial issue in your family?	
Yes.	10.1
No.	89.9
11. Are you in the first generation of your family to attend college?	
Yes	5.1
No	94.9
12. If either or both of your parents attended BYU, did either or both of them study a major within or related to the life sciences (e.g., biology, microbiology, zoology, health, nursing, etc.)?	
a. Yes	15.7
b. No	55.8
c. Neither of my parents attended BYU	28.5
13. Are either or both of your parents in a life science profession? (e.g., Doctor, dentist, nurse, research scientist in the life sciences, etc.)	
a. Yes	18.1
b. No	81.9
14. How much do you think your parent’s opinion of evolution has influence your own views on the subject?	
Very strongly influenced.	13.4
Somewhat influenced	42.2
Influenced very little.	44.4
15. How much do you think your parents’ opinion about evolution has been influenced by their own educational experience?	
Very strongly influenced.	15.5
Somewhat influenced	57.1
Influenced very little.	27.4
16. Was the teaching of evolution a controversial issue in your community of state?	
Yes	32.6
No.	67.4

Responses are from Cohort 2 (2014–2016).

With regard to preparatory science instruction, 31% report strong coverage of evolution from earth science or various biology courses in secondary school (62% said the coverage was weak, 6% report it was absent; Questions 1 & 2). Although some recall a degree of bias on the part of their high school biology teacher (about equal numbers pro and con religious concerns), most (63%) were neutral (Questions 3). About 17% accepted the science they were taught without conflict, nearly 36% felt some degree of religious conflict, and the same number report a personal sense of neutrality (Questions 4).

With regard to anti-evolution pronouncements from Church authorities, sixty-one percent said evolution was not discussed in their LDS seminary (high school religious instruction) classes (Questions 5), but 23% said that when it was they came away with a feeling that the science and their religion were compatible. These students generally did not perceive evolution as a controversial issue growing up. Ninety percent said it was not an issue in their family (Questions 10), and 67% said it was not an issue in their community (Question 16),

Only a small number, 13%, report having heard of, or read, the book *Man His Origin and Destiny* [24], arguably the most widely known anti-evolution LDS publication of the 20^th^ century, and only half had heard about, or read, *Mormon Doctrine* [31], whose coverage of evolution is similarly condemnatory (Questions 6 &7). Only 8% had recently heard a negative expression from an ecclesiastical leader about the subject (Question 9). Fifty-five percent believe their own views on evolution have been influenced by their parents’ opinions (Questions 14), which in turn were influence by their prior academic experience (73%; Question 15).

#### Comparison of BYU legacy and non-legacy students

To test the third causal mechanism, C) the BYU legacy effect, students were asked whether or not their parents attended BYU (an item in the pre-instruction survey not reported in Table 2). The survey shows that respondents represent a high proportion of legacy admissions–those whose parents previously studied at BYU. For fathers, 56% graduated and another 6% attended; for mothers the numbers are 48% and 15%. Twenty-eight percent of the sample were not BYU legacies. To determine the effect of legacy, we calculated separate tabulations of survey results for current students who are BYU legacies (whose parents are represented by Cohort 1), and the 28% who are not. The results for four relevant items are shown in Table 3. For the non-legacy subgroup, there is an 18% reduction in the degree to which their own or their parents’ views of evolution were strongly or somewhat strongly influenced by the latter’s educational experience (Questions 1 & 2; X^2^(1) = 6.30, *p* = 0.01, X^2^(1) = 6.06, *p* = 0.01, respectively). Non-legacy students, however are equally likely to correctly perceive that the LDS Church does not have an official position on evolution (Questions 3; X^2^(1) = 2.14, *p* = 0.14). Interestingly, the non-legacy students are more likely to perceive a conflict between science and revealed religion, although the difference did not reach significance (Questions 4; X^2^(1) = 3.42, *p* = 0.06).

**Table 3:** Survey responses of BYU legacy and non-legacy students.

Survey Item	Legacy (%)	Non-Legacy (%)
Was your parents’ opinion about evolution strongly or somewhat strongly influenced by their own educational experience?	77.2	59.7
Has your parents’ opinion about evolution strongly or somewhat strongly influenced your own views on the subject/	58.4	40.0
There is no official position of the LDS Church concerning evolution.	75.0	64.5
The concepts of science are frequently in conflict with the revealed word of God.	11.9	22.8

Responses are from Cohort 2 (2014–2016).

### Research Question 3: Can strategic instruction based on reconciliation increase student acceptance?

#### Post-instruction essay

**Fig 2:**
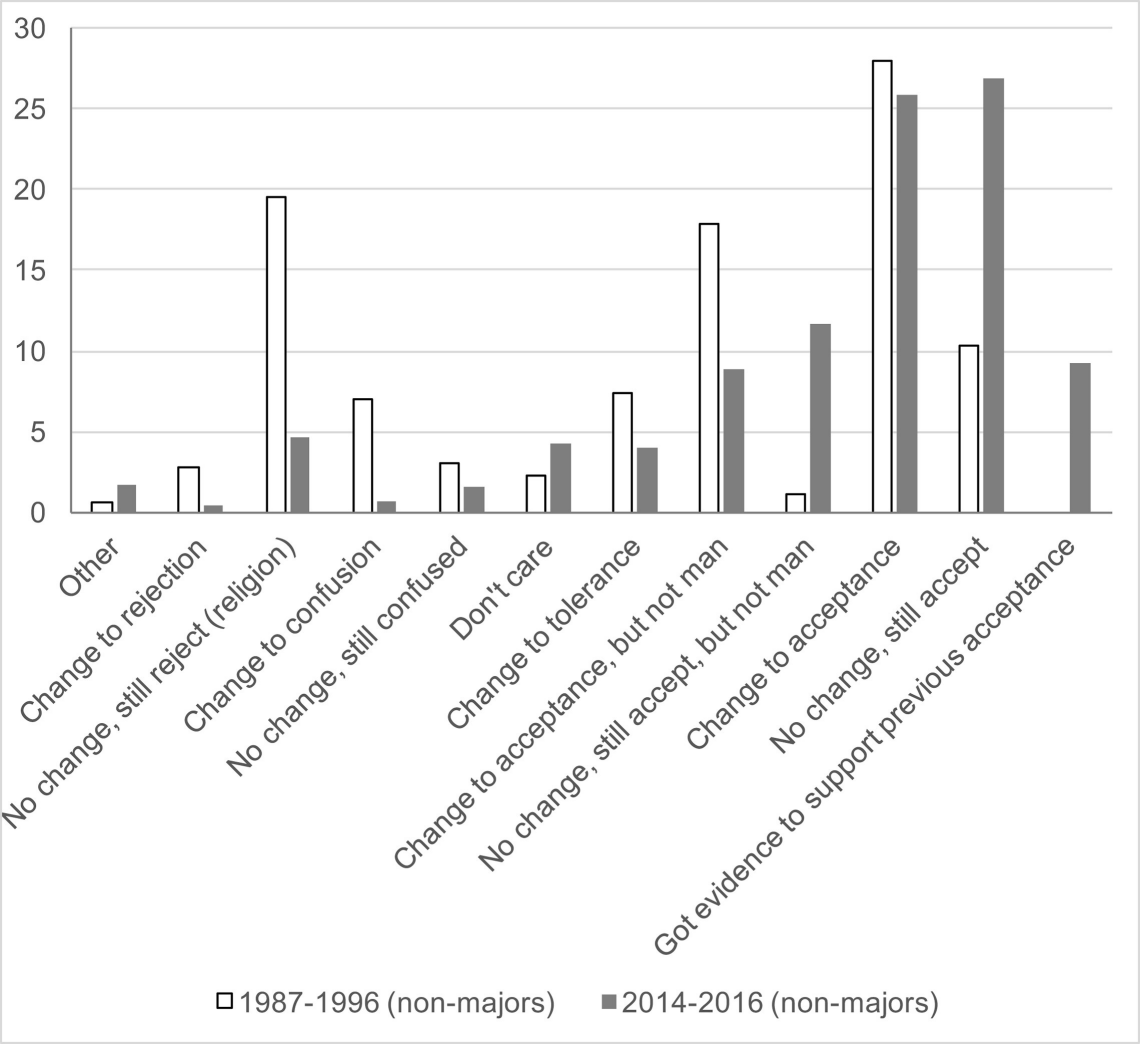
Post-essay comparison in non-majors biology. This is a comparison of the distribution of the themes of change (or no change) of the post-essays following a semester in the non-majors biology courses (Biol. 100) between 1987–1996 (light bars) and between 2014–2016 (dark bars). Themes are abbreviated and ordered, approximately, from least accepting (left) to most accepting (right).

To answer our third research question, whether a one-semester course in biology covering evolution with an attempt at reconciliation causes a more immediate shift in acceptance, a second essay was required following the in-class presentation of principles of evolution (concepts and evidentiary examples). Students were instructed to reconsider their pre-instruction essay, and indicate if any change in opinion had occurred and if so, the reasons why. A comparison of students from both Cohorts is displayed in Fig 2 (see list of themes constituting the coding rubric in S2 File). The results for Cohort 1 demonstrate that the course coverage produced a significant shift in the direction of positive attitudes. [Comparing the percentage of students who fully accept evolution between the pre- and post-essays, X^2^(1) = 15.93, *p* < 0.001]. For example,

“Before this semester I knew very little about the theory of evolution. It had always seemed like a far-fetched idea and an excuse for people to deny the existence of God. After having studied a little about evolution, I have been able to form my opinion about the theory. First I learned that I should not be so quick to discount a theory without having studied the facts. I also learned that evolution is not always pitted against religion. I am grateful that I have had the opportunity to learn about evolution without feeling like my own personal religious beliefs were under attack. In fact, my faith in my God is stronger now after this experience.”

Overall acceptance (31%) or with the exception of humans (18%) were the largest categories, with 15% still expressing religious conflict.

The same dramatic, pre/post positive shift toward acceptance of evolution exhibited by Cohort 1 was exhibited by those in Cohort 2 [again, comparing those with full acceptance from pre- to post-essay, X^2^(1) = 8.28, *p* = 0.004]. Twenty-six percent changed their views toward acceptance. Twenty-six percent retained an original affirmative perspective (more than 2-fold that of Cohort 1). There was a 10% reduction in those who maintained an anti-evolution perspective because of religious conflict. About 9% additionally noted that the course had provided compelling evidence for their initial opinion that evolution was a valid principle. For example,

“Not much has changed in my personal views about the subject of evolution. I still certainly believe as before that evolution has happened and is still happening not only with animals but with humans as well. This class has, however, caused me to have a greater understanding on the subject of evolution. So my beliefs that I had before taking the class were only strongly supported throughout the evolution [section of] this class, and further convinced me of those beliefs. [cites: phylogenetic tree relationships, DNA homologies, hominid fossils, Galapagos finches].”

Many students in Cohort 2 commented on the importance of learning that the LDS Church does not, in fact, have an official position on the validity of evolutionary theory. For example,

“In addition to the scientific evidence, it was also helpful to read the words of church leaders regarding both sides of the evolution question and learn that the church does not have an official position on evolution and that previous statements were simply statements of opinion, not official church doctrine.”

### Research Question 4: Is a shift in acceptance of evolution an indicator of changing beliefs about science in general?

#### Pre-instruction survey–nature of science

To test our fourth research question, that this shift in acceptance of evolution is indicative of a shift in views toward science, we asked students specific questions about science and technology as reported in Table 4. Question 1 offers the same options regarding evolution presented in the Gallop Poll [12], and reveals a strong religious influence when the focus is on humans. Fifty-five percent chose the creationist position; 43% opted for a God-guided human evolution over millions of years, and 3% for the same without divine intervention. Forty percent approve of the teaching of Intelligent Design in a biology classroom, but 53% reported no opinion (Question 2). Seventy-four percent disapprove of parents not vaccinating their children (Question 3). Fifty percent accept the reality of climate change, but 29% have no opinion (Question 4).

**Table 4:** Views about scientific concepts and the impact of technology.

Question	Response (%)
1. Which of the following statements comes closest to your views on the origin and development of human beings?	
a. Human beings have developed over millions of years from less advanced forms of life, but God guided this process.	42.9
b. Human beings have developed over millions of years from less advanced forms of life, but God had no part in this process.	1.8
c. God created human beings pretty much in their present form at one time within the last 10,000 years or so.	55.3
2. What is your opinion about teaching the concept of Intelligent Design in a biology course?	
a. I approve.	39.5
b. I disapprove.	7.2
c. I have no opinion on this issue.	53.3
3. What is your opinion about parents who do not vaccinate their children because they believe vaccination may lead to autism?	
a. I agree with such parents.	2.5
b. I disagree with such parents.	74.3
c. I have no opinion on this issue	23.2
4. What is your opinion on the issue of climate change?	
a. Climate change is real; the earth is warming due to human activity.	52.3
b. There are no valid data suggesting that climate change is real	18.9
c. I have no opinion on this issue.	28.8
5. Because of their professional emphasis on reason and empirical observation, it is generally more difficult for people trained in science to maintain religious faith.	
a. True.	29.1 (*40*)
b. False.	70.9 (*60*)
6. The concepts of science are frequently in conflict with the revealed word of God.	
a. True.	14.7 (*41*.*1*)
b. False.	85.3 (*58*.*9*)
7. The technological and scientific advances of the past centuries have changed the world. There are many negative consequences of this, however, because the changes wrought by science are often unnatural. For example, synthetic fertilizers, refined food, food preservatives, and insecticides are not part of the natural world and are incompatible with it. As a result, mankind suffers.	
I agree.	29.4 (*24*.*7*)
I disagree.	70.6 (*75*.*3*)
8. Extensive testing by the United States government has shown the drug Laetrile (an extract from apricot pits) to be ineffective in the cure of cancer. The drug cannot be legally used in the United States; it is legal in Mexico. Do you agree with the following statement? “the ban on Laetrile should be lifted. People should be allowed to exercise their own agency in using the material, even though tests have proven it is without value?	
I agree.	37.8 (*32*.*9*)
I disagree.	62.2 (*67*.*1*)
9. It is immoral to tamper with human genes be altering, adding to, or eliminating part of the complement of DNA which an individual has inherited over generations by natural means.	
I agree	51.6 (*47*.*6*)
I disagree	48.4 (*52*.*4*)
10. The United States Patent and Trademark Office has ruled that higher life forms–even mammals–produced by genetically engineering DNA sequences may be patented. Industrial leaders are pleased and see the ruling as helping to protect their investments. Critics express anxiety about the safety and morality of tempering with life forms. Do you agree that commercial companies ought to be able to patent such organisms?	
Yes.	26.3 (*26*.*4*)
No.	73.7 (*73*.*6*)

Responses are from Cohort 2 (2014–2016). In Questions 5–10, the italicized numbers in parentheses are the average values for 10,774 students in the course in Cohort 1 (1987–1996).

Questions 5–10 of the survey are identical to items presented to Cohort 1, permitting an instructive comparison. Fewer current students (29% cf. 40%) see an empirical epistemology as antithetical to religious faith, although the difference was no significant (Question 5; X^2^(1) = 2.17, *p* = 0.14). Significantly fewer (15% cf. 41%) view scientific principles to be in conflict with revealed religious tenets (Question 6; X^2^(1) = 16.04, *p* < 0.001). Most in both cohorts (70–75%) do not view the products of modern technology in a negative light (Question 7; X^2^(1) = 0.35, *p* = 0.56) and approve patenting of genetically modified organisms (Question 10; X^2^(1) = 0.00, *p* = 1.00). On the issue presented in Question 8, legal access to the compound Laetrile, 38% of current students come down on the side of patient choice (compared to 33% earlier), in spite of the empirical evidence (X^2^(1) = 0.33, *p* = 0.56). Students in both cohorts are evenly split on the issue of whether the practice of modifying human DNA is immoral (Question 9; X^2^(1) = 0.18, *p* = 0.67).

#### Post ecology and environment survey

To serve as an internal control for changing beliefs in science in general, and to further indicate that the trends we are seeing are specific to evolution, we administered a survey in both cohorts on prominent controversial environmental issues. Attitudes about environmental issues are shown to be more stable over the same 20-30-year interval, as shown by the results of questionnaire responses recorded in Table 5. Question 1 documents a modest reduction (about 10%) in support of environmental organizations, and nearly one-fifth currently express no interest (X^2^(4) = 5.10, *p* = 0.28. There was about the same distribution of views about the major environmental problems (Questions 2, X^2^(4) = 2.54, *p* = 0.88). Conspicuously absent from the questionnaire, given current concerns, is climate change/global warming, which was not perceived as an issue by the general public at the time of the earlier sample. About twice as many students currently see a limit to the human population (Question 3, X^2^(4) = 11.90, *p* = 0.01), but a continued number (approximately one-third) who are undecided or unconcerned about this issue. No change was observed in views about wilderness areas (Question 4; X^2^(4) = 0.99, *p* = 0.94), oil and gas shortages (Question 5, X^2^(4) = 4.51, *p* = 0.35), or the conflict between extinction of endangered species and economic development (Question 6, X^2^(4) = 2.30, *p* = 0.74).

**Table 5:** Environment questionnaire (percent response) for each cohort for a representative sample of the questions.

Question	‘87-‘96	2014–16
1. Which of the following best represents your personal view about the environmentalist movement?		
a. Environmental organization, such as the Sierra Club, provide a valuable service in helping protect the environment.	68.3	58.3
b. Governmental agencies (federal and state) are the only organizations needed to protect the environment.	8.4	7.6
c. Local governments can adequately handle environmental crises.	3.8	7.4
d. Environmental organizations, in general, have caused more problems than they have solved by preventing governmental agencies from performing their duties, by hampering growth and development, and by preventing sportsmen from utilizing game species.	9.4	7.4
e. I have no interest in the environmental movement.	10.1	19.3
X^2^(4) = 104.94, *p* < .001		
2. Which of the following do you consider to be the major environmental problem of the current decade?		
a. The loss of wilderness areas.	20.4	23.8
b. Air and water pollution.	69.2	59.6
c. The extinction of species.	3.5	6.4
d. Growth of the human population.	4.8	6.9
e. There are no major environmental problems in the current decade. I am tired of hearing about environmental problems. Besides, economic problems are more important than environmental issues.	2.1	3.3
X^2^(4) = 46.37, *p* < .001		
3. Which of the following best represents your personal view about the growth of the human population?		
a. The earth has a carrying capacity and there is a limit to the number of humans that can be supported.	26.9	47.7
b. There is no limit to how many humans can be supported on this earth. Modern technology will supply the necessary food and energy.	22.4	15.8
c. We should be concerned with human population growth in underdeveloped countries. In such countries, family size should be limited.	16.2	6.1
d. Growth should be limited in all countries.	2.2	1.3
e. None of the above.	32.3	29.1
4. Which of the following best represents your personal view about preserving certain locations in the country as wilderness areas.		
a. The concept of wilderness areas is valid, but the areas presently designated as wilderness areas are inadequate.	46.1	46.2
b. The concept of wilderness areas is valid. The balance between wilderness and developed land is currently adequate.	37.2	41.0
c. Wilderness areas should be reduced to those areas enclosed within national park boundaries.	3.6	2.4
d. Wilderness areas as now defined should be eliminated. These areas should be opened up to grazing, all-terrain vehicles, etc.	1.8	0.7
e. None of the above.	11.3	9.7
5. Which of the following best represents your personal view about the possibility of future oil and gasoline shortages?		
a. There is no real oil shortage. Oil companies circulate false information and manipulate supplies in order to drive up prices and increase their profits.	6.8	2.8
b. There are large reserves of fossil fuel in the earth which have not yet been tapped. These will meet every conceivable energy need, worldwide, for hundreds or thousands of years to come.	8.5	12.9
c. Engineering technology will come to our aid and provide synthetic fuel alternatives (or other scientific solutions) which will meet our future needs and permit us to maintain our present levels of energy consumption.	30.8	43.1
d. The oil shortage is real. Gasoline prices are likely to rise dramatically within a few years and a realistic projection of the not too distant future is a reduction in the oil-dependent lifestyle of people in industrial nations.	42.4	30.8
e. I have no opinion on this issue.	11.5	10.5
6. Which of the following best represents your personal view about conflicts between a biological interest (preserving from extinction a rare species of fish) and economic development (building a dam or a road)?		
a. Economic development has the highest priority. Mankind was given dominion over all the earth and we have greater value than lesser animals. Fish are expendable if they stand in the way of progress.	4.6	6.9
b. Such conflicts are seldom if ever real. The biological interests are not based on real data and their importance is blown out of proportion. Ecologists are generally obstructionists who should be disregarded most of the time.	4.3	3.0
c. Humans should consider their species an integral part of an ecosystem and realize their dependence on other organisms. Compromises should be sought (even if there is a higher cost, for example, by rerouting a road to a less convenient location).	75.3	73.4
d. Preserving endangered species has the highest priority. Man's stewardship of the earth requires us to resist the extinction of any life form, an event which must be considered an unnatural, unnecessary tragedy.	8.8	5.6
e. I am completely neutral on this issue.	7.0	11.0

## Discussion

In regards to our first research question, the data reported above support the clear conclusion that there has been a positive shift in attitudes toward evolution by recent LDS undergraduates at BYU. Examples include acknowledgment of the reliability of the fossil record and other geological evidence, acceptance of the deep age of the earth, and a correct understanding of theory in a scientific context. Compared to the generation of 20–30 years ago, current students are much more accepting. Interestingly, many of the responses to the survey questions about ecology and the environment remain relatively unchanged over the study period, serving as something of an internal control that validates the evolution data. If anything, there appears to be less interest in environmental protection and greater support for industrial development. We attribute this result to the highly partisan (Republican) perspective of many in the LDS community.

Additional validation of this conclusion comes from the content of written commentaries whose themes feature intellectual openness to the validity of the scientific data, and little concern about conflict with religious values. The concept no longer generates the disbelief and apprehension that it did in years past. Generally speaking, the LDS Church has espoused conservative positions on social and political issues. Over the past 100 years, members of the church have predominantly held an anti-evolution opinion on the question of the origin of human-kind, even in the absence of an official declaration about the modus operandi of creation. From essay responses, this appears to have changed for the younger generation of students.

We have also provided evidence (pre-post survey data) that strategic classroom instruction has contributed to this shift. The anecdotal experience of many current instructors, whose courses include a presentation of evolutionary theory, is that there is very little resistance by those enrolled. Efforts to achieve reconciliation of the science and students’ religious conviction are usually successful. Most appear to welcome the invitation to be believers in both realms of inquiry.

The analyses provided above also suggest plausible potential explanations for these longitudinal changes in attitude. Survey responses confirm that students come to BYU with better high school instruction in science, and that they have been exposed to far fewer negative messages from their religious community. Moreover, we have shown that prior positive instruction about evolution received by their parents has had a salutary effect. The explanation for the change in perception about evolution is undoubtedly complex and will vary somewhat from individual to individual. From our analyses, we suggest three potentially highly influential factors responsible for this shift in attitudes, and provide further elaboration below.

### Prior exposure to principles of evolution (K-12 preparation)

In the last 30 years there have been concerted efforts on a national level to improve pre-college education in evolutionary science. An appraisal of their high school science courses by current BYU students seems to reflect this improved coverage of evolution. Only 9% report that the subject was not included in the curriculum, and 37% appraised the coverage as strong (Table 2). The contents of essays by these students (Cohort 2; Fig 1) also contain references to canonical examples and validating arguments for evolution acquired from their K-12 education. This seems to be reflective of these national efforts to improve science education. Tests generated by the College Board, including CLEP, SAT, and AP have included items on fundamentals of evolution, and have therefore driven the inclusion of the subject in the curriculum of nearly all school districts. For example, a mandatory audit of AP courses requires certain evolution standards before they can receive certification from the College Board [47]. In addition, in 2008 the National Academy of Sciences produced a booklet, *Science*, *Evolution*, *and Creationism* [48], containing a very helpful summary of the evidence and arguments supporting evolution with arguments countering the anti-evolution position.

The State of Utah, from which 35% of BYU students are drawn, has been a leader in promoting the inclusion of evolution in the science classroom. The Utah State Board of Education Science Core Standard in Biology relative to evolution, updated in 2003 and readopted in 2012 for grades 9–12, reads as follows [49]:

STANDARD 5: Students will understand that biological diversity is a result of evolutionary processes.

Objective 1: Relate principles of evolution to biological diversity.
Objective 2: Cite evidence for changes in populations over time and use concepts of evolution to explain these changes.
Objective 3: Classify organisms into a hierarchy of groups based on similarities that reflect their evolutionary relationships.

The details listed below each of the three Objectives are comprehensive and consistent with the current accepted principles of the subject.

In 2005 the Utah State Board of Education approved, and the Utah Science Teachers Association adopted, the “Utah State Board of Education Position Statement on Teaching Evolution” [50]. This strongly worded endorsement of evolution as a primary unifying concept in science defined and defended the concept and urged sensitivity by teachers in relating to the belief systems of students. Many of those responsible for this pro-evolution movement are former and current BYU faculty members who continue to work in a partnership with school district administrators, school boards, and state education officers in implementing sound science education standards in the state [51].

Nevertheless, knowledge of evolution does not appear to be equivalent to acceptance of evolution. Studies have demonstrated that religious objections to evolution are resistant to change, even after thorough instruction in the subject. A particularly difficult religious hurdle is the “essentialism” notion, that intrinsic characteristics that define different organisms (the Biblical “kinds”) cannot be altered; only adaptations within major groups are tolerated [52]. It has also been demonstrated ([53], p.665) that “the socializing agent of religion outweighs the effect of education on attitudes related to evolution,” and that partisanship is also a key predictor of opinion. Both of these factors are relevant in the LDS community; the teaching of evolution in public schools remains controversial to a degree [54]. We would argue, however, that young Mormons are quite willing to accept evolutionary theory as valid when they are helped to accommodate the science into a doctrinal framework that acknowledges a degree of theological ignorance about matters of creation, and demonstrates that the Church does not have an official position on the subject.

### A reduction in condemnatory pronouncements

In the period from 1970–1990 there were a large number of written statements and public addresses to LDS audiences whose content was directed at questioning the validity of evolution and suggesting that it was a harmful idea. This included utterances by General Authorities of the Church in official conferences or publications (e.g., [55–57]) and in talks given on the campus at BYU (e.g. [33, 34]). Books with a similar intent directed at an LDS audience also appeared in about this same time frame (e.g., [55–60]). The overall effect of these communications, we believe, was to perpetuate in the Mormon community the strongly anti-evolution sentiment first articulated in the late 1950s [30, 31].

In contrast, very little of this negative rhetoric has appeared in the last 25 years (but see [61]). In support of the conclusion that there has been a reduction in negative messaging, current BYU students are significantly less aware of the writings of Smith and McConkie (Table 2) than students a generation earlier. Nearly 70% report that the teaching of evolution was not a controversial issue in their communities, and 91% report that it was not controversial in their families. It is highly significant that 64% report that evolution was not discussed in their church seminary instruction. Likewise, there are anecdotal reports from several who now (or formerly) teach religion classes at BYU, that negative messaging from that quarter has subsided drastically in recent years (Personal Communications). The beginning of a reduction in a science versus religion conflict probably coincides with the production of the “BYU Evolution Packet,” mentioned previously. Only 9.5% of current students, however, report knowledge of this collection. In 2005 that original document was replaced in the BYU library by a more complete volume [Evenson/Jeffery, 42] explaining the origin and content of the “Packet.” In the 11 years since this book appeared in the library at BYU, 9 copies of it were circulated only 390 times–a very small number in a student body of greater than 30,000 (Personal communication. Circulation Department Harold B. Lee Library, BYU; electronic access may have been higher.) Interestingly, the authorized student manual for the Church’s Old Testament course [62] whose contents have not been changed since 1980, continues to present an anti-science, narrowly creationist perspective authored by a Seventh-Day Adventist professor [63]. Generally, however, the BYU community, and to some extent in the LDS world at large, evolution has lost much of the unsavory reputation and disapproval it once held.

Notably there is a large body of publications by LDS authors validating evolution and its compatibility with religious doctrines (especially since 1970). In addition, there is a large faculty group in the College of Life Sciences performing research directly related to evolution and evolution education (e.g., [64–69]). However, we have no evidence that these have been a major force in influencing the views of current students. Rather, they are probably generally unknown (references to any of these works were conspicuously absent from the personal essays written by current students).

### A generational influence

Regarding the generational legacy, a significant portion (72%) of students now matriculating at BYU come from parents who also attended the university. Only 16% of these parents were majors in the life sciences (meaning that the majority would have enrolled in Biology 100 in order to fulfill the General Education requirements for graduation), and only 18% were subsequently involved as a life science professional. This university course, then, would have been their primary source for information about, and a positive attitude toward, evolution. Approximately 15,000 students in the earlier period were exposed to that educational philosophy in Biology 100 at BYU. Many others, who were majors in the biological sciences received the same message. It is possible, that as a result, current students report that the teaching of evolution was not an issue in their families, and that their own views on the subject were shaped in large part by the attitudes of their parents, which was influenced in turn by their own academic backgrounds. We submit, then, that there is a strong correlation between change in acceptance and legacy status suggesting that perhaps greater open mindedness about this aspect of science has been transmitted from the earlier generation to their offspring.

### The impact of strategic instruction

The three factors just elaborated (K-12 instruction, a reduction in negative messaging, and the positive impact of a parental generation) would all be expected to affect their influence on students before they participated in biological coursework. The generational legacy, we submit has potentially been mediated through the instruction in biology experienced by their parents at BYU. Pre-post data from both the earlier and current periods demonstrate a dramatic increase in acceptance of evolution by the end of the Biology 100 course, from 12.7% to 38.3% in Cohort 1 and from 39.4% to 75.4% in Cohort 2. The very low initial acceptance rate in Cohort 1 is a reflection of the prevailing negative sentiment that existed at that earlier time in much of the Church membership outside of the academy. It was manifested, on occasion, through letters sent to church headquarters and to the university administration raising objections to the affirmation of evolutionary biology by the science faculty (Personal Communications). The current generation shows much less of this sentiment. We share here some of the pedagogical strategies that may have promoted this outcome as examples of how to implement change in a highly conservative, religious community.

Many scientists have approached the teaching of evolution based on a “Deficit Model”–the view that a lack of acceptance was the result of ignorance, remedied by more detailed coverage [70, 71]. An alternative, the “Resolution Model” suggested that the conflict with religion could be settled in favor of science by heavy emphasis on empirical evidence, absent from religious concepts [72–75]. As cited earlier [52], there is reason to question the effectiveness of these strategies.

We would characterize our approach as a “Reconciliation Model,” through which students are able to allow coexistence between their scientific and religious perspectives. Although some aspects of the LDS context may be unique (e.g. the very high regard for the pronouncements of ecclesiastic authorities and a doctrine that encourages all LDS to get as much education in any field of study as is feasible), we believe our approach may be relevant to those of other faiths. The following are examples of proposals to which students are exposed, set in a constructivist framework, that can serve as a guide for instructors at any university interacting with religious students:

- Evolution is not a belief system and therefore does not have to be in conflict with religion (evolution is not inherently atheistic).
  1. Many religious organizations are supportive of evolutionary science, e.g., [72]
  2. There are examples of respected scientists who are persons of faith.
  3. Biblical passages can often be appropriately interpreted figuratively (e.g. the age of the earth).
  4. Students are encouraged to determine exact doctrines of their religions towards evolution rather than relying on cultural precedence.
- Science cannot prove or disprove religion.
  1. The evolutionary explanation for life’s origins does not require a role for deity, but there are no data excluding that possibility.
  2. Intelligent design is a religious, and not a scientific, construct.
- The evolutionary explanation for humankind is not demeaning to human dignity.
  1. The diversity of living things produced by evolution is awe-inspiring.
  2. There is no evidence that acceptance of evolution is linked to social evils such as racism, juvenile delinquency, or drug addiction.
  3. Our primitive hominin ancestors had complex societies and behaviors that make it difficult to determine boundaries between hominin species, including our own.
- Much of the conflict results from misconceptions.
  1. We share a common ancestor with chimpanzees but are not “descended from” chimpanzees or “monkeys”.
  2. A theory, in scientific terms, is not the same as a theory, in common vernacular.
  3. The correct evolutionary metaphor is a tree, not a ladder.
  4. Natural selection permits speciation, not just adaptation within “kinds.”
  5. Organisms do not evolve because of “decisions” based on need.
  6. The Lamarckian concept of the inheritance of acquired characteristics is not valid.

Our reconciliation approach has also been highlighted as a way of being culturally competent in instructing students with a strongly religious background [26]. We submit that this approach has contributed to the shift toward acceptance demonstrated above. It is part of a concerted effort to present the scientific fundamentals as being compatible with religious faith, with emphasis on the LDS Church’s lack of any official doctrinal position on the subject. This has clearly had a significant impact in reducing tension about the possible negative consequences of the subject for many individuals, and helped to facilitate a reconciliation of their religious and scientific belief systems.

### Shifts in views on the nature of science dealing with controversies

It is possible that favorable views about evolution documented here translate into a generalized acceptance by our students of science as a valid epistemology. This would be manifest through other science-related questions with a similar approval by this sample. Alternatively, the views of beginning students about publicly controversial issues may each be influenced by a unique set of factors independent of their scientific content. Our data suggest the latter. For example, the 40% approval by our students for inclusion of Intelligent Design into the biology classroom is, at least in part, an appeal to the notion of “fairness”, i.e. “People have a right to hear both sides of the question, don’t they?” Careful analysis in this instance demonstrates that fairness does not apply–ID is a religious-based concept, not a scientific alternative to evolution. On another issue, nearly one-fourth of our students express “no opinion” of the matter of parental choice of vaccination for their children. While the science in this case is absolutely clear, it is confused by celebrity advocacy of non-vaccination and countered by the view that parental choice should be honored in a decision that is personal. Again, careful analysis of the potentially dire public health consequences of failure to vaccinate demonstrates that this is not just a private, personal consideration [7].

Certainly, there is the potential impact of partisan politics. Mormons are disproportionately Republican, and Republicans disproportionately discount both evolution and climate change. The data reported here for evolution run counter to this trend, but the views expressed on environmental concerns probably do reflect this influence. About half of our students doubt the reality of human caused climate change or have no opinion about it. These individuals may see the issue in conservative economic terms, i.e. “There are immediate negative consequences of regulation of CO_2_ emissions, weighted against less certain future projections; by comparison, evolution happened in the distant past and is a finished process.” There is also a difference in the nature and accessibility of the evidence. For climate change the data are relatively abstruse; reliance on the word of experts is required. In contrast, the visual evidence of fossils is tangible and much less subject to question.

These considerations lead us to conclude that from an educational perspective, each of these controversial issues needs to be studied independently because beliefs about each are influenced by factors separate from the scientific data. In general, for practical matters such as advances in medicine, transportation, and communication, Latter-day Saints accept and applaud science and technology, and in fact, view these benefits as divine blessings which further the Church and it mission. This was certainly the view of Church President Hinckley in a General Conference address to church members ([76], p.88):

[The twentieth century] has been the best of all centuries. … The life expectancy of man has been extended by more than twenty-five years. Think of it. It is a miracle. The fruits of science have been manifest everywhere. By and large, we live longer, we live better. This is an age of greater understanding and knowledge. … This has been an age of enlightenment. The miracles of modern medicine, of travel, of communication are almost beyond belief.

Given this approval of science in general, where, then does the LDS Church and its members fit along the spectrum of belief with respect to evolution, biology’s most foundational principle? We note that this question must be addressed at several different levels (doctrinal, ecclesiastic, cultural, and academic) and the answer is not the same for each. The Church is doctrinally neutral, meaning neither in favor (endorsement) nor opposed (rejection), as evidenced by a recent statement in a Church publication: “The Church has no official position on the theory of evolution. Organic evolution, or changes to species’ inherited traits over time, is a matter for scientific study. Nothing has been revealed concerning evolution” [77]. But there is a significant degree of residual hostility among some leaders and some members of the Church at large. Awareness of this attitudinal diversity is very useful to science teachers of LDS students and others with a similar religious background.

## Conclusion

The data shown here reveal a shift toward acceptance by LDS youth of evolutionary theory as a valid explanation for current life on earth. While human evolution is still something of an issue, there has been a dramatic decrease in conflict in comparison with the views of college students of two to three decades ago. As explanations, the data support the influence of an improved K-12 educational experience, a reduction in negative messaging from people in authority within the religious organization, and the positive effects of a strong BYU science education in a faith-friendly manner, which we suggest can be transmitted as a generational legacy. Students both previous and current have responded with approval of both the theoretical framework and empirical evidence for evolution when presented in an instructional strategy that clarifies the authoritative position of their church and encourages reconciliation of faith and science. These data are specific to those of the LDS faith, some aspects of which may be unique (i.e. close adherence of the membership to authoritative pronouncements of any current church leadership, a unity of belief and standardized religious practice in congregations world-wide, and highly effective programs of religious education for youth at both the family and ecclesiastical levels). On the other hand, a case can be made for generalization. The BYU population represents students of a Christian faith whose teachings are informed by Biblical scripture (including the precept of divine creation), with notorious cultural (but not doctrinal) barriers to evolution, a case similar to many other conservative religions. As a result, we believe our findings can be applied more broadly, used as an “ecological case study,” whose results can be replicated with students of other religious affiliations. As “nothing in biology makes sense except in the light of evolution” [78], the fact that trends are changing among one of the most resistant populations in America should serve as a point of encouragement for life science educators from all institutions.

## Supporting information

S1 File Pre-Essay prompt and rubric. This is the essay prompt given to students at the beginning of the semester. Also included is an example of the types of statements used to categorize essays.
(DOCX)

S2 File Post-Essay prompt and rubric. This is the essay prompt given to students at the end of the semester to determine whether change occurred. Explanations of each category are given.
(DOCX)
