# Supplementary material for: A longitudinal study of attitudes toward evolution among undergraduates who are members of the Church of Jesus Christ of Latter-day Saints
Source: PLoS One. 2018 Nov 7;13(11):e0205798. doi: 10.1371/journal.pone.0205798 (PMC6221276; doi:10.1371/journal.pone.0205798)
Supplement: S1 File — This is the essay prompt given to students at the beginning of the semester. Also included is an example of the types of statements used to categorize essays. (DOCX) [file pone.0205798.s001.docx]

**S1 Pre-Essay Prompt and Rubric**

**Evolution Essay Prompt #1**

Your assignment is to write an approximately 500-word statement that expresses your personal sentiments about evolution.  Do not study the text or read other sources on the subject; this is not a research paper.  We're interested in reading your current, individual opinion.  What is your concept of evolution?  What are the words, images, or feelings that come to you when you hear that word? After reading your statement we should be able to know clearly what evolution means to you:  how you define it, what you know or don't know about it, whether it evokes positive or negative feelings, and anything else that is relevant to your point of view.  After you've described what that view is, explain how you've come to that position.  What are your sources of information?  Who's influenced you?

**Evolution Essay #1 Rubric**

The responses were coded as belonging to one of the following categories, depending on the major theme expressed in each.

Category

1. Discomfort. “I have a generalized discomfort, fear, or confusion about biological evolution.
2. Demeaning. “Evolution is demeaning (man’s relatedness to low forms is a repugnant, offensive idea”.
3. Improbable. “ Evolution is improbable (a ludicrous idea – nonsense- which could not possibly be true”.
4. Religious conflict. “I don’t believe in evolution because it’s in conflct with religious views I hold (or have been taught).”
5. Avoidance. “I avoid the subject. I associate it with controversy or negative feelings, and mentally tune out.”
6. Theory. “Evolution is only a theory (a “2^nd^ class” concept which probably contains serious flaws)”.
7. Man Different. “Man is unique. Evolution might apply to lower forms, but not to man.”
8. Ignorance. “I’m basically ignorant about the concept of evolution, and don’t have an opinion. Maybe I should try to learn more about it.”
9. Equivocal. “I believe in evolution, yet I don’t. some of the evidence seems compelling, some does not.” Or, “Evolution can explain the physical, but is inadequate in providing a satisfying total view of man.”
10. Suspended judgment. “Resolving the matter is not a high priority matter for me.”
11. Acceptance. “ Evolution is a valid concept reflecting the origin and relatedness of life forms.” Or, “I can reconcile evolutionary ideas with my theological understanding.”
12. Adaptation Only. “There might be adaptation, but not evolution across major animal boundaries.”
