# Supplementary material for: A longitudinal study of attitudes toward evolution among undergraduates who are members of the Church of Jesus Christ of Latter-day Saints
Source: PLoS One. 2018 Nov 7;13(11):e0205798. doi: 10.1371/journal.pone.0205798 (PMC6221276; doi:10.1371/journal.pone.0205798)
Supplement: S2 File — This is the essay prompt given to students at the end of the semester to determine whether change occurred. Explanations of each category are given. (DOCX) [file pone.0205798.s002.docx]

**S2 Post-Essay Prompt and Rubric**

**Evolution Essay Prompt #2**

The subject of this 300-500 word statement (about 4 paragraphs) is *evolution*. Your task is to evaluate your personal views about the subject as a result of having spent one-fourth of the semester studying it. Think about what you wrote in the Opinion Statement at the beginning of the semester. How do you feel now in comparison to the way you felt at the beginning of the semester? Carefully defend your present point of view. If there has been a change, describe it, and then attempt to explain the reasons for your new perspective.

**Evolution Essay #2 Rubric**

The responses were coded as belonging to one of the following categories, depending on the major theme expressed in each.

Category

1. In comparison to Opinion Statement #1, a change toward acceptance of evolution as a valid concept.
2. In comparison to Opinion Statement #1, a change toward acceptance, with the exception of humans.
3. In comparison to Opinion Statement #1, a change toward greater confusion or discomfort.
4. In comparison to Opinion Statement #1, a change toward rejection of evolution as a valid concept.
5. In comparison to Opinion Statement #1, a change to tolerance of a different point of view.
6. No change from Opinion Statement #1; still accept evolution as valid.
7. No change from Opinion Statement #1; accept with the exception of humans.
8. No change from Opinion Statement #1; still reject, primarily because of religious conflict.
9. No change from Opinion Statement #1; still confused.
10. Don’t really care about the issue; it’s unimportant.
11. My initial acceptance has been validated with compelling evidence.
12. Other miscellaneous responses that do not fit into any of the above categories.
